# Influence of MnZn Ferrite Homogeneous Fibers on the Microstructure, Magnetic, and Mechanical Properties of MnZn Ferrite Materials

**DOI:** 10.3390/ma16010209

**Published:** 2022-12-26

**Authors:** Yajing Shang, Fan Luo, Zhongxia Duan

**Affiliations:** Institute of Electrical Engineering, Chinese Academy of Sciences, Beijing 100190, China

**Keywords:** MnZn ferrite homogeneous fibers, homogeneous-fiber-reinforced MnZn ferrite, microstructure, magnetic properties, mechanical properties

## Abstract

MnZn ferrite homogeneous fibers were synthesized via a simple solvothermal method and they were used as a reinforcing phase to prepare homogeneous-fiber-reinforced MnZn ferrite materials. The effects of MnZn ferrite homogeneous fibers (0 wt% to 4 wt%) doping on the microstructure, magnetic, and mechanical properties of MnZn ferrite materials were studied systematically. The results showed that MnZn ferrite homogeneous fibers exhibited high purity, good crystallinity, and smooth 1D fibrous structures, which were homogeneous with MnZn ferrite materials. Simultaneously, a certain content of MnZn ferrite homogeneous fibers helped MnZn ferrite materials exhibit more uniform and compact crystal structures, less porosity, and fewer grain boundaries. In addition, the homogeneous-fiber-reinforced MnZn ferrite materials possessed superior magnetic and mechanical properties such as higher effective permeability, lower magnetic loss, and higher Vickers hardness compared to ordinary MnZn ferrite materials. In addition, the magnetic and mechanical properties of homogeneous-fiber-reinforced MnZn ferrite materials first increased and then gradually decreased as the homogeneous fiber content increased from 0 wt% to 4 wt%. The best magnetic and mechanical properties of materials were obtained as the fiber content was about 2 wt%.

## 1. Introduction

MnZn ferrite is an important member of the soft magnetic materials, which has been widely used in network communication, information storage, automatic control, medical diagnosis, and various apparatuses from household appliances to scientific equipment [1,2,3]. Moreover, it has superior characteristics such as high magnetic permeability, high electrical resistivity, high stability, large magnetic induction, and low magnetic loss [4,5,6]. Thus, it is crucial to develop MnZn ferrite exhibiting better performances with the rapid development of modern science and technology.

After decades of studies, it is well known that doping a small amount of oxides is one of the most effective strategies for improving the performances of MnZn ferrite. Common oxides doped in MnZn ferrite have been systematically researched and can be divided into three types: First, dopants such as SiO_2_ [7], CaO [8], and HfO_2_ [9] tend to change the chemical compositions and further adjust the resistivity of grain boundaries. The first kind of dopants can enhance the resistivity and lower the loss of MnZn ferrite, but the segregation of the above cations at the grain boundaries may bring down the initial permeability of the material due to magnetic dilution. Second, soluble oxides involving TiO_2_ [10], SnO_2_ [11], NiO [12], and Co_2_O_3_ [13] are easy to dissolve into the lattices and alter the performance. The second kind of dopants can effectively adjust the intrinsic magnetic properties of MnZn ferrite. However, they may also reduce partial magnetic properties of the material. Third, dopants with low melting points such as V_2_O_5_ [14], Bi_2_O_3_ [15], and MoO_3_ [16] can modify the microstructure of the MnZn ferrite during the process of sintering. The third kind of dopants is critical for improving the permeability and reducing the loss of MnZn ferrite. Larger amounts of non-magnetic dopants will damage the microstructure and reduce the properties of the material. Accordingly, it can be found that the addition of the above dopants can improve some performances of MnZn ferrite, but often lead to the deterioration of other performances. Therefore, the MnZn ferrite homogeneous-fiber- and particle-co-reinforced structure is proposed to prepare the MnZn ferrite with excellent microstructure, magnetic, and mechanical properties.

In this work, MnZn ferrite homogenous fibers synthesized by the solvothermal method were used as the reinforcing phase to prepare homogeneous-fiber-reinforced MnZn ferrite materials. The microstructure, phase composition, magnetic, and mechanical properties of materials were characterized by scanning electron microscopy (SEM), X-ray diffraction (XRD), an LCR meter, a magnetic automatic test device, and a hardness tester. Furthermore, the effect of homogeneous fiber content (0 wt% to 4 wt%) on the homogeneous-fiber-reinforced MnZn ferrite materials was investigated in detail.

## 2. Experimental Details

### 2.1. Synthesis of MnZn Ferrite Homogenous Fibers

MnSO_4_·4H_2_O (≥99%), ZnSO_4_·7H_2_O (≥99%), FeSO_4_·7H_2_O (≥99%), H_2_C_2_O_4_ (≥98%), ethylene glycol (EG, ≥99%), and deionized water were used as raw materials. In a typical process, 0.5 mmol MnSO_4_·4H_2_O, 0.5 mmol ZnSO_4_·7H_2_O, and 2 mmol FeSO_4_·7H_2_O were dissolved in a mixture of EG and deionized water with a volume of 30 mL (EG:water = 3:1). Then, 3 mmol H_2_C_2_O_4_ was added into the above mixture under continuous stirring for 60 min. Subsequently, the obtained mixture was transferred into a Para polyphenyl-lined autoclave of 50 mL capacity and heated at 120 °C for 24 h. After that, the products were centrifuged to separate solids, washed with ethanol and deionized water for several times, and dried in an oven at 80 °C for 12 h. Finally, the as-prepared precursor fibers were annealed in air atmosphere at 500 °C for 2 h. The MnZn ferrite homogenous fibers, as a nominal composition of Mn_0.5_Zn_0.5_Fe_2_O_4_, were collected by the solvothermal method.

### 2.2. Synthesis of Homogeneous-Fiber-Reinforced MnZn Ferrites

MnO (≥99%), ZnO (≥99%), Fe_2_O_3_ (≥99%), and MnZn ferrite homogenous fibers (Mn_0.5_Zn_0.5_Fe_2_O_4_, made by our laboratory) were used as raw materials. The preparation process was as follows: First, MnO, ZnO, and Fe_2_O_3_ were evenly mixed for 120 min at a speed of 300 r/min in the planetary mill. After that, the evenly mixed powders were dried at 90 °C for 10 h in the oven. Subsequently, the dried powders were pre-sintered at 850 °C for 2.5 h in an air atmosphere and cooled to room temperature. Next, the pre-sintered materials were evenly mixed for 120 min at a speed of 300 r/min, and then dried at 90 °C for 10 h. Additionally, the dried materials were mixed with 0.85 wt% polyvinyl alcohol for granulation, followed by 50 mesh screening to obtain the MnZn ferrite powders. Then, the as-prepared MnZn ferrite homogenous fibers of 1 wt%, 2 wt%, 3 wt%, and 4 wt%, together with MnZn ferrite powders, were evenly mixed and dried to obtain the 1 wt%, 2 wt%, 3 wt%, and 4 wt% homogeneous-fiber-reinforced MnZn ferrite composite powders. After that, the composite powders were pressed into toroidal shapes with the dimensions of OD (outer diameter) 20 mm, ID (inner diameter) 10 mm, and h (height) 8 mm under an external load of 350 MPa for 15 min. Finally, the annular samples were sintered at 1280 °C for 6 h in a Blank equilibrium atmosphere-controlled sintering system to obtain the required 1 wt%, 2 wt%, 3 wt%, and 4 wt% homogeneous-fiber-reinforced MnZn ferrite samples. In addition, the ordinary MnZn ferrite sample was prepared with 0 wt% MnZn ferrite homogeneous fibers by the above preparation process.

### 2.3. Characterizations

The surface morphology of the samples was characterized by SEM (Supra 35VP, ZEISS, Jena, Germany). The phase composition and crystallographic structure of the samples were analyzed by XRD (D8 Advance, Bruker, Karlsruhe, Germany) over the 2θ range of 10° and 80° with Cu Kα irradiation (λ = 0.15418 nm) at 40 kV and 40 mA. The LCR meter (IM3570A988-06, HIOKI E.E. CORPORATION, Hioki, Japan) was used to measure the effective permeability of the samples at room temperature with a frequency range of 10 kHz to 1000 kHz. The magnetic automatic test device (MATS-3000SA, Hunan Linkjoin Technology Co., Ltd., Loudi, China) was used to measure the magnetic loss of the samples at room temperature in the frequency range from 10 kHz to 1000 kHz. The Vickers hardness of the samples was determined on a hardness tester (HV-1000ZDT, Shanghai Jvjing Precision Instrument Manufacturing Co., Ltd., Shanghai, China). The load was 200 g and the loading time was 15 s.

## 3. Results and Discussion

### 3.1. Characterization of the MnZn Ferrite Homogeneous Fibers

**Figure 1:**
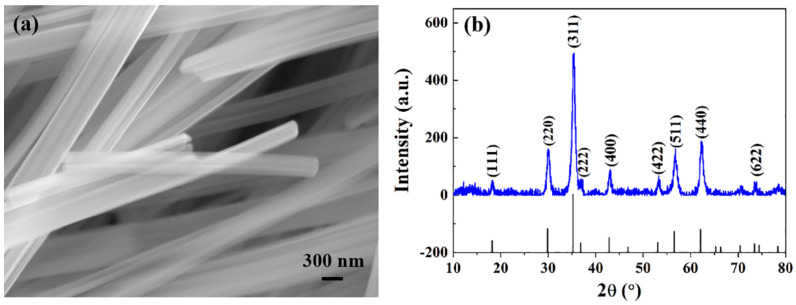
(**a**,**b**) SEM image and XRD spectrum of the MnZn ferrite homogeneous fibers.

The general SEM image of the as-prepared MnZn ferrite homogeneous fibers is shown in Figure 1a. It can be seen that the typical MnZn ferrite homogeneous fibers exhibited a 1D fibrous structure with a large aspect ratio and had a smooth surface area. The XRD pattern of the corresponding MnZn ferrite homogeneous fibers is shown in Figure 1b. It can be observed that the major diffraction peaks appearing at 18.1°, 29.8°, 35.1°, 36.7°, 42.6°, 52.9°, 56.3°, 61.8°, and 73.3° were assigned to the (111), (220), (311), (222), (400), (422), (511), (440), and (622) planes, respectively, and matched well with the standard powder diffraction data of MnZn ferrite, which indicated the high purity and good crystallinity of MnZn ferrite homogeneous fibers. In addition, the MnZn ferrite homogeneous fibers had the same chemical composition as the MnZn ferrites. Therefore, it can be known that the MnZn ferrite homogeneous fibers prepared by the solvothermal method had high purity, good crystallinity, and smooth 1D fibrous structures, which were homogeneous with MnZn ferrites.

### 3.2. Microstructure Characterization Analysis of MnZn Ferrite

**Figure 2:**
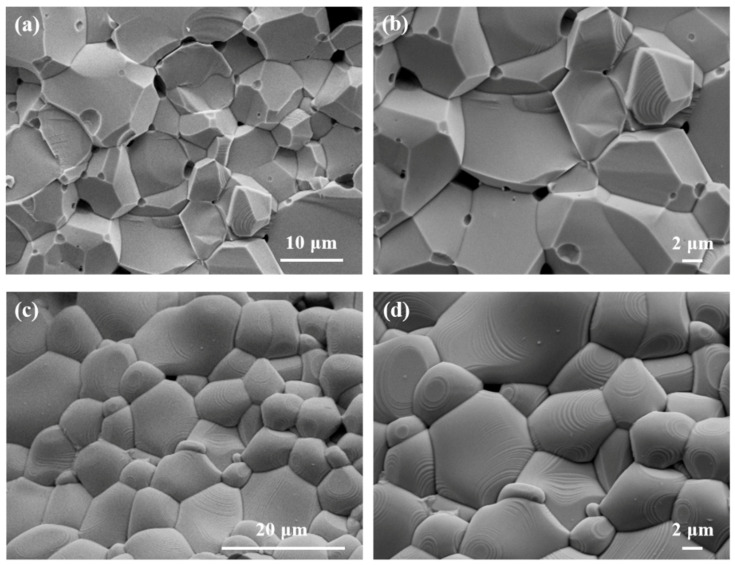
SEM images of typical MnZn ferrite samples. (**a**,**b**) 0 wt% ordinary MnZn ferrite; (**c**,**d**) 2 wt% homogeneous-fiber-reinforced MnZn ferrite.

The SEM images of typical MnZn ferrite samples are shown in Figure 2. Figure 2a,b display a cross-sectional morphology of ordinary MnZn ferrite with 0 wt% MnZn ferrite homogeneous fibers. Figure 2c,d reveal a representative cross-sectional morphology of homogeneous-fiber-reinforced MnZn ferrite with 2 wt% MnZn ferrite homogeneous fibers. It can be clearly seen from Figure 2a,b that the 0 wt% ordinary MnZn ferrite sample had a relatively good crystal structure, while there were small pores between the grains of the sample. With the introduction of MnZn ferrite homogenous fibers, it can be noticed from Figure 2c,d that the 2 wt% homogeneous-fiber-reinforced MnZn ferrite sample had a more uniform and compact crystal structure. Meanwhile, the sample exhibited less porosity and fewer grain boundaries.

This was because the introduction of MnZn ferrite homogenous fibers took the bridge between the grains, which could induce grain crystallization and promote the homogeneity of the materials. In addition, the nanoscale MnZn ferrite homogeneous fibers with finer grain size than the MnZn ferrite particles could make the composite powders rearrange at the low-temperature sintering stage, and the homogeneous fibers can be filled into the small pores between the grains, gradually eliminating the pores and grain boundaries of materials. Thus, the homogeneous-fiber-reinforced MnZn ferrite exhibited a better microstructure compared with the ordinary MnZn ferrite.

### 3.3. Magnetic Properties Analysis of the Homogeneous Fiber Reinforced MnZn Ferrite

**Figure 3:**
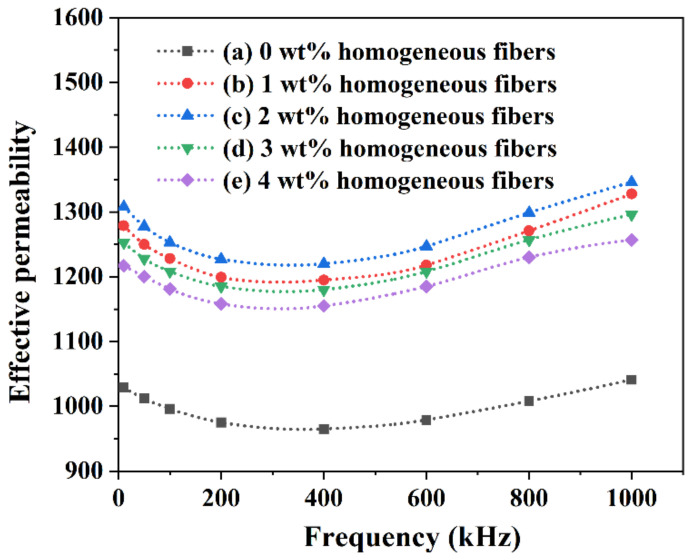
Effective permeability of the homogeneous-fiber-reinforced MnZn ferrite samples versus frequency at room temperature: (a) 0 wt%, (b) 1 wt%, (c) 2 wt%, (d) 3 wt%, and (e) 4 wt%.

To understand the function of MnZn ferrite homogeneous fibers on the magnetic properties of the homogeneous-fiber-reinforced MnZn ferrites, the effective permeability and magnetic loss of the homogeneous-fiber-reinforced MnZn ferrite samples prepared with different fiber contents (0 wt% to 4 wt%) were measured by an LCR meter and magnetic automatic test device, respectively. The effective permeability of the homogeneous-fiber-reinforced MnZn ferrite samples with different fiber contents (0 wt% to 4 wt%) versus frequency at room temperature is shown in Figure 3. It can be seen that the effective permeability of all samples first decreased and then gradually increased with the increase in the frequency range from 10 kHz to 1000 kHz. At the same frequency, the effective permeability of the 1 wt%, 2 wt%, 3 wt%, and 4 wt% homogeneous-fiber-reinforced MnZn ferrite samples was higher than that of the ordinary MnZn ferrite sample with 0 wt% homogeneous fibers. In addition, it can be found that the effective permeability of the homogeneous-fiber-reinforced MnZn ferrite samples increased with the increase in fiber content as the content of MnZn ferrite homogeneous fibers was less than 2 wt%, and then gradually decreased with the increase in fiber content as the fiber content was larger than 2 wt%. The effective permeability reached a maximum as the fiber content was 2 wt%.

It can be known that the internal grain size and grain interface structure of MnZn ferrite materials affected the dynamic balance during the magnetization process [17]. The permeability was directly proportional to the average grain sizes and inversely proportional to the number of grain boundaries and pores [18]. Ordinary MnZn ferrite with 0 wt% homogeneous fibers had more pores and grain boundaries than the homogeneous-fiber-reinforced MnZn ferrites, so the effective permeability was relatively low. As the homogeneous fiber content increased, the crystal grains gradually grew and became uniform, so the pores were gradually eliminated and the effective permeability gradually increased. When the homogeneous fiber content exceeded 2 wt%, owing to the fact that the fibers deposited at grain boundaries and impeded domain wall motion, the permeability decreased.

**Figure 4:**
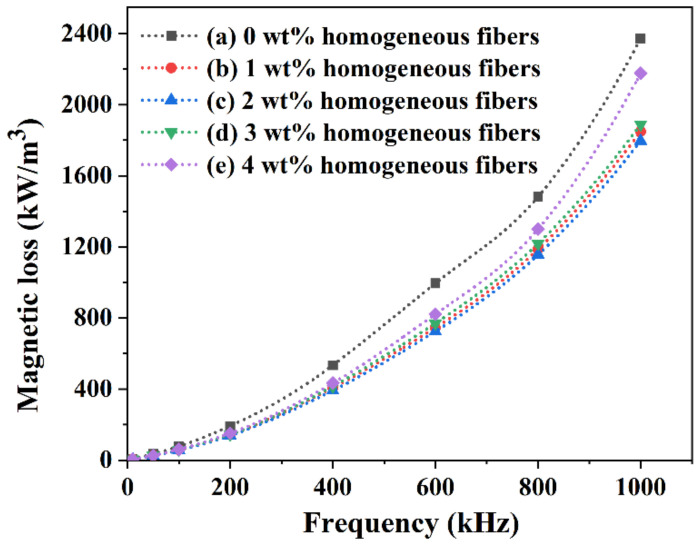
Magnetic loss of the homogeneous-fiber-reinforced MnZn ferrite samples versus frequency at room temperature: (a) 0 wt%, (b) 1 wt%, (c) 2 wt%, (d) 3 wt%, and (e) 4 wt%.

Figure 4 shows the magnetic loss of the homogeneous-fiber-reinforced MnZn ferrite samples with different fiber contents (0 wt% to 4 wt%) versus frequency at room temperature. It can be found that the magnetic loss of all samples increased gradually with the increase in frequency. At the same frequency, the magnetic loss of the homogeneous-fiber-reinforced MnZn ferrite samples with the fiber content from 1 wt% to 4 wt% was lower than that of the ordinary MnZn ferrite sample with 0 wt% homogeneous fibers. In addition, the magnetic loss of the homogeneous-fiber-reinforced MnZn ferrite samples first decreased and then gradually increased with the rise in the homogeneous fiber contents from 1 wt% to 4 wt%. The minimum magnetic loss was obtained as the fiber content was 2 wt%.

The cause of this phenomenon was that the MnZn ferrite with 2 wt% homogeneous fibers had a uniform and compact crystal structure, which could effectively restrain the interaction between magnetic particles and greatly reduce the magnetic loss. Thus, a certain content of MnZn ferrite homogenous fibers was beneficial to improve the magnetic properties of MnZn ferrite materials. Meanwhile, the homogeneous-fiber-reinforced MnZn ferrite materials exhibited the best magnetic properties as the fiber content was about 2 wt%.

### 3.4. Mechanical Properties Analysis of the Homogeneous-Fiber-Reinforced MnZn Ferrite

**Figure 5:**
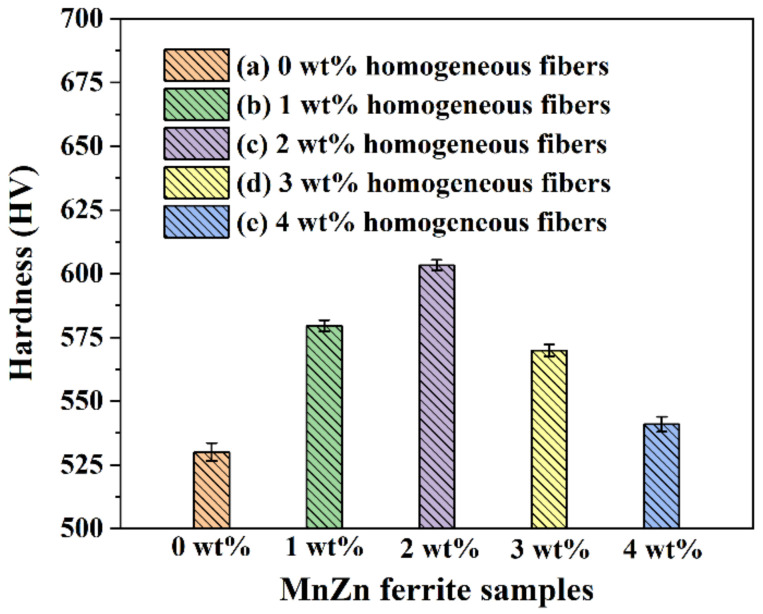
Vickers hardness of homogeneous-fiber-reinforced MnZn ferrite samples with different fiber contents: (a) 0 wt%, (b) 1 wt%, (c) 2 wt%, (d) 3 wt%, and (e) 4 wt%.

To study the impact of the MnZn ferrite homogeneous fibers on the mechanical properties of the homogeneous-fiber-reinforced MnZn ferrites, the Vickers hardness of the homogeneous-fiber-reinforced MnZn ferrite samples synthesized with different fiber contents (0 wt% to 4 wt%) was measured by the hardness tester. Figure 5 shows the Vickers hardness of the homogeneous-fiber-reinforced MnZn ferrite samples with different fiber contents (0 wt% to 4 wt%). Meanwhile, the Vickers hardness values for seven groups of repetitive tests of the homogeneous-fiber-reinforced MnZn ferrite samples with different fiber contents (0 wt% to 4 wt%) are shown in Table 1. As we can see, the average Vickers hardness values of 0 wt%, 1 wt%, 2 wt%, 3 wt%, and 4 wt% homogeneous-fiber-reinforced MnZn ferrite samples were 530.1, 579.6, 603.3, 585.1, and 540.9 HV, respectively. The corresponding standard deviations of the samples were 3.49, 2.16, 2.06, 3.06, and 2.96 HV. It can be seen that the deviations in Vickers hardness for the samples were within a certain range. The results indicated that the 1 wt%, 2 wt%, 3 wt%, and 4 wt% homogeneous-fiber-reinforced MnZn ferrite samples had a much higher Vickers hardness than the 0 wt% ordinary MnZn ferrite sample. At the same time, the Vickers hardness of the corresponding homogeneous-fiber-reinforced MnZn ferrite samples first increased and then gradually decreased with the increase in homogeneous fiber contents from 1 wt% to 4 wt%. In addition, the maximum Vickers hardness was obtained as the fiber content was 2 wt%, which was consistent with the test results of magnetic properties.

It is known that good crystal structure is the guarantee of materials with high magnetic and mechanical properties. For the homogeneous-fiber-reinforced MnZn ferrite materials, the MnZn ferrite homogeneous fibers were used as the reinforcing phase to construct a co-reinforced structure of homogeneous fibers and particles. In this co-reinforced structure, the homogeneous fibers could effectively weaken the damage caused by stress concentration, so the particles could improve the microregional stress distribution of materials [19,20]. In the crystallization process, the added homogeneous fibers could bridge between the original particles, providing more paths for the diffusion of atoms during sintering, strengthening the reaction and crystallization kinetic conditions. In addition, the addition of fibers had an impact on the movement of dislocations on the matrix. The fibers were obstacles for the dislocation movement, and internal stresses also occurred. Therefore, the hardness of MnZn ferrite materials increased. More importantly, the MnZn ferrite homogeneous fibers had the same chemical composition, perfect lattice matching, and good surface wettability with the MnZn ferrite particles [21,22]. It can completely overcome the problem of unstable interface structures between heterogeneous fibers and particles, due to their different thermal conductivity, thermal expansion coefficient, elastic modulus, and Poisson’s ratio. Accordingly, the strength and stability of homogeneous-fiber-reinforced MnZn ferrite materials were enhanced. Thus, doping a certain content of MnZn ferrite homogenous fibers gave an optimized crystal structure of MnZn ferrite materials, improving the strength and stability of materials. Meanwhile, homogeneous-fiber-reinforced MnZn ferrite materials exhibited the best mechanical properties as the fiber content was about 2 wt%.

## 4. Conclusions

In this paper, we put forward a MnZn ferrite homogeneous-fiber- and particle-co-reinforced structure and revealed the effect of MnZn ferrite homogeneous fibers (0 wt% to 4 wt%) doping on the microstructures as well as magnetic and mechanical characteristics of MnZn ferrite materials. The result showed that MnZn ferrite homogeneous fibers prepared via the solvothermal method exhibited high purity, good crystallinity, and smooth 1D fibrous structures, which were homogeneous with MnZn ferrite materials. In addition, the homogeneous-fiber-reinforced MnZn ferrite materials had a denser crystal structure, less porosity, and fewer grain boundaries, exhibiting excellent properties of high permeability, low magnetic loss, and high Vickers hardness in comparison to 0 wt% ordinary MnZn ferrite materials. Simultaneously, the magnetic and mechanical properties of the homogeneous-fiber-reinforced MnZn ferrites first increased and then gradually decreased with the rise in the homogeneous fiber contents from 1 wt% to 4 wt%. The optimal content of the homogeneous fibers was around 2 wt%.

## Figures and Tables

**Table 1:** Vickers hardness values for seven repetitions of homogeneous-fiber-reinforced MnZn ferrite samples with different fiber contents: (a) 0 wt%, (b) 1 wt%, (c) 2 wt%, (d) 3 wt%, and (e) 4 wt%.

Samples	Vickers Hardness (HV)							Average Value (HV)	Standard Deviation (HV)
	1	2	3	4	5	6	7		
(a) 0 wt%	525.2	526.7	529.7	528.2	531.2	534.3	535.3	530.1	3.49
(b) 1 wt%	580.1	579.0	577.8	583.6	578.4	581.3	576.7	579.6	2.16
(c) 2 wt%	606.6	601.6	605.3	601.6	600.4	603.5	604.1	603.3	2.06
(d) 3 wt%	567.6	569.8	573.2	566.5	572.1	571.5	568.1	585.1	3.06
(e) 4 wt%	542.1	537.4	545.2	540.5	541.5	543.6	536.3	540.9	2.96

## Data Availability

Not applicable.
